# Using QTL mapping to investigate the relationships between abiotic stress tolerance (drought and salinity) and agronomic and physiological traits

**DOI:** 10.1186/s12864-015-1243-8

**Published:** 2015-02-05

**Authors:** Yun Fan, Sergey Shabala, Yanling Ma, Rugen Xu, Meixue Zhou

**Affiliations:** University of Tasmania, P.O. Box 46, Kings Meadows, TAS 7249 Australia; Barley Research Institution of Yangzhou University, Yangzhou, 225009 China

**Keywords:** QTL analysis, Drought tolerance, Proline content, Leaf wilting, Salinity tolerance

## Abstract

**Background:**

Drought and salinity are two major abiotic stresses that severely limit barley production worldwide. Physiological and genetic complexity of these tolerance traits has significantly slowed the progress of developing stress-tolerant cultivars. Marker-assisted selection (MAS) may potentially overcome this problem. In the current research, seventy two double haploid (DH) lines from a cross between TX9425 (a Chinese landrace variety with superior drought and salinity tolerance) and a sensitive variety, Franklin were used to identify quantitative trait loci (QTL) for drought and salinity tolerance, based on a range of developmental and physiological traits.

**Results:**

Two QTL for drought tolerance (leaf wilting under drought stress) and one QTL for salinity tolerance (plant survival under salt stress) were identified from this population. The QTL on 2H for drought tolerance determined 42% of phenotypic variation, based on three independent experiments. This QTL was closely linked with a gene controlling ear emergency. The QTL on 5H for drought tolerance was less affected by agronomic traits and can be effectively used in breeding programs. A candidate gene for this QTL on 5H was identified based on the draft barley genome sequence. The QTL for proline accumulation, under both drought and salinity stresses, were located on different positions to those for drought and salinity tolerance, indicating no relationship with plant tolerance to either of these stresses.

**Conclusions:**

Using QTL mapping, the relationships between QTL for agronomic and physiological traits and plant drought and salinity tolerance were studied. A new QTL for drought tolerance which was not linked to any of the studied traits was identified. This QTL can be effectively used in breeding programs. It was also shown that proline accumulation under stresses was not necessarily linked with drought or salinity tolerance based on methods of phenotyping used in this experiment. The use of proline content in breeding programs can also be limited by the accuracy of phenotyping.

**Electronic supplementary material:**

The online version of this article (doi:10.1186/s12864-015-1243-8) contains supplementary material, which is available to authorized users.

## Background

Drought and salinity are two major abiotic stresses that severely limit agricultural production worldwide. The severity and occurrence of both drought and salinity stresses is expected to increase as a result of global environmental changes, causing major implications for food supply [1,2]. Compounding this is an increasing world population that requires a rise in food production by more than 70% before 2050 [3]. Some sustainable and economical solutions to achieve this goal is to develop more drought-tolerant and salt-tolerant crops [4]. However, very slow progress has been made in improving tolerance, or developing tolerant cultivars, due to the physiological and genetic (quantitative inheritance) complexity of tolerance traits. Also, the high variability of the field environments and the low efficiency of selection methods further handicap the progress. Most researchers agree that it is highly unlikely that tolerance to these stresses may be improved by manipulating the expression level (function) of only one gene [5,6]. More likely, we should brace ourselves for a painstakingly slow pyramiding of useful traits. Taking salinity stress tolerance as an example, vacuolar Na^+^ sequestration mediated by the NHX Na^+^/H^+^ exchanger [7] could be not possible without a sufficient activity of the tonoplast H^+^-pump to energize this process [2]. Moreover, this sequestration will become a futile cycle if Na^+^ back-leak from the vacuole via Na^+^-permeable fast (FV)- and slow (SV)- vacuolar channels is not prevented [8,9]. Given that the molecular identity of some of these transport systems (e.g. FV channels) is yet to be revealed, a transgenic approach to such pyramiding remains highly challenging.

MAS technology implies the use of a set of markers which are closely linked with the target gene(s) for an indirect selection of a specific trait without phenotyping the trait. While great progress has been achieved in using MAS approach for crop breeding under a range of stresses where the tolerance is conferred by one or two major genes, the progress has been more modest when it comes to either salinity or drought tolerance. Numerous physiological and morphological traits were used as indirect selection criteria for both salinity and drought tolerance. Leaf wilting, relative water content (RWC) and proline contents are among the most frequently used for drought tolerance [10-12]. Physiological and biochemical responses used as selection criteria for salinity tolerance include: seed germination under stress conditions, relative water content, wet and dry weight of roots and shoots, chlorophyll content, shoot sodium content, plant survival, tissue proline and carbohydrate content [13-15].

Proline is a widely distributed osmolyte which protects plants against drought and salinity [16]. It is mainly synthesized from glutamate by two enzymes: pyrroline-5-carboxylate synthetase (P5CS) and pyrroline-5-carboxylate reductase (P5CR) [17]. Apart from acting as an osmolyte to balance osmotic pressure in cells, proline also plays important roles in regulating cellular reactive oxygen species (ROS) balance [18,19], cell signalling and plant development such as rapid cell division, floral transition and embryo development [20]. Proline was also shown to be able to affect intracellular ionic homeostasis by controlling ion transport across cellular membranes [21,22]. Proline levels increase dramatically in plants under both drought [23] and salinity [24] conditions, and it was repeatedly suggested that using high proline levels as a biochemical marker may benefit stress breeding programs (reviewed in [25]). However, higher proline levels were also found in drought-hypersensitive [26,27] and salinity-susceptible genotypes [28,29], and the causal relationship between proline accumulation and stress tolerance in plants is not as straight forward as initially thought.

In a natural environment, drought and salinity stresses are often combined [30]. Both drought and salt stress trigger cellular dehydration and cause osmotic stress which then leads to cytosolic and vacuolar volume reduction [31,32]. Abiotic stress such as cold, drought and salt stress are controlled by many common and conserved regulatory pathways [33,34]. Drought tolerance QTL influenced growth under salt stress by reducing salt uptake [35], indicating that some QTL/genes may have pleiotropic effects on multi-stress tolerance.

Both drought and salinity tolerance are quantitatively inherited and controlled by several genetic loci. While many QTLs have been reported for drought [36-39] and salinity tolerance [14,15,40,41], very few of the linked markers have been successfully used in breeding programs due to the relatively lower heritability of the QTL and other factors affecting the gene expression. The success of using physiological traits as indirect selecting criteria for both drought and salinity tolerance relies on the true correlations between these traits and the tolerance. Most studies used very few varieties to study the relationships between drought/salinity tolerance and different agronomic/physiological traits or simply mapping QTLs for different traits under drought or salinity stress [10,37,39,42], which may not necessarily reflect the tolerance genes. This issue was overcome in this work by using a doubled haploid (DH) population: 1) to investigate the linkage between various agronomic and physiological traits and drought and salinity tolerance, and 2) to identify QTLs controlling tolerance to these two stresses in barley.

## Methods

### Plant material

A total of 72 F_1_-derived doubled-haploid (DH) lines generated from a cross between TX9425 and Franklin were used in this study. TX9425 is a Chinese landrace, two-rowed barley, variety which also exhibits some particular agronomic traits [43] and disease resistance [44]. Franklin is an Australian two-rowed malting barley - regarded as salinity sensitive variety [40,45].

### Evaluation of drought tolerance and relevant physiological traits

Three separate experiments were conducted for evaluating drought tolerances; each experiment was repeated three times.

*Experiment I and II*: five seeds of parental varieties and DH lines were sown in large containers (1.6 m × 2.5 m × 0.6 m) filled with a pine bark/loam-based potting mix with a premixed 6–9 month slow release Osmocote fertiliser incorporated. Both trials were arranged as a randomized complete block design with two replications in each trial. The containers were located in a glasshouse with controlled temperature (day/night, 25/16 ± 2°C) at the Mt Pleasant Laboratories in Launceston, Tasmania, under natural light. Trials were conducted in 2012/13 and 2013/14 growing seasons. The trials were kept watered in early growth stage, using an automatic irrigation system (spraying from the top). From early tillering stage, the watering stopped in half of the containers, with the latter half left drying. When the most susceptible lines showed severe symptoms of wilting (approximately ×four weeks after drought treatment, Additional file 1: Figure S1), the scoring of wilting was conducted (Exp I and II) and the second fully expanded leaves (Exp II) were sampled for the evaluation of proline content. Scores of 1 – 10 was used where the score of 1 = very tolerant and the score of 10 = very sensitive (Additional file 1: Figure S1).

*Experiment III*: each parent varieties or DH lines were sown in 2-L pots filled with potting mixture. All the pots were placed in six different trays, each contained a whole replication. The trial was arranged as a randomized complete block design with three replications for both the control and the drought treated. The water level was kept 2–3 cm high (i.e. 2–3 cm water at the bottom of the pots) in the tray. Half of the trays were kept dry starting from the early stage of tillering. Similar to Exp I and II, when the most susceptible lines showed severe symptoms of wilting, the scoring of wilting was conducted and the second fully expanded leaves were sampled for the evaluation of proline content. The first and second fully extended leaves from different plants were sampled for measuring moisture content.

### Evaluation of salinity tolerance and relevant physiological traits

Seeds of parental varieties and the DH lines were sown in large plastic containers (1.6 m × 0.8 m × 0.6 m) filled with a pine bark/loam based potting mixture with a premixed slow release fertiliser. Each genotype comprised of three replicates, each of five seeds. Controls were omitted in this case, since it has been shown in our earlier report, that different varieties or DH lines grown in the same potting mixture, but with no salt added, showed no apparent symptoms of leaf chlorosis or dead leaves [40]. The salt treatment was similar to that previously described [15,40]. Salt stress was started at the three-leaf stage. A solution containing 320 mM NaCl was used to wash through the tanks several times until the solution draining out from the tanks had a consistent salt concentration. The treatment was repeated every three days. When the most susceptible lines showed severe symptoms, salt tolerance was assessed by combining scores for leaf chlorosis and plant survival when most of the DH lines reached booting stage (0 = no damage and 10 = all dead) [15]. The second leaves of the DH lines were collected for proline assay and top two leaves from different plants were collected for measuring Na^+^ contents.

### Measurement of Na^+^ content in leaves and relative moisture content

Fresh leaves were weighed soon after collection. The samples were dried in a 60°C oven for two days and dry weights were then recorded. Moisture content was calculated from the difference between fresh weight and dry sample weights. For the Na content, leaf sap were extracted and centrifuged at 5000 rpm for 10 min as described elsewhere [46]. The supernatants were collected to evaluate Na^+^ content using a flame photometer.

### Measurement of proline content

Proline content was estimated according to the method of Mittler [47] and Sayed et al. [36]. Leaf samples were collected and ground to fine power. 30 mg of leaf power was homogenized in 2 ml of 3% sulphosalicylic acid (SA), vortexed and then centrifuged at 4000 rpm for 10 min. 500 μL of the supernatant was taken into a glass tube and 500 μL 3% SA was added, followed by the addition of 1 mL ninhydrin acid and 1 mL glacial acetic acid. The homogenate was heated at 100°C for 1 hour in a water bath, and then quickly cooled in the ice bath. 2 ml toluene was then added to each tube and vibrated for 30 sec. Tubes were kept at room temperature for at least 10 min to allow phase separation until the bottom layer became clear. The absorbance of the upper layer with toluene was read at 520 nm. Proline content was determined by a standard curve from known concentrations of L-proline. The proline content in control samples was not detectable for the dilutions used in this method; hence, only proline content under drought and salinity stress are presented.

### QTL analysis

A molecular map of this population has been published earlier [48]. The genetic linkage map was constructed using 412 DArT markers, 80 AFLP markers and 28 microsatellite markers. The average distance between markers = 2.12 cM but markers were not evenly distributed among Chromosomes with some gaps being greater than 20 cM. Significant marker distortion was also observed in some regions on all seven chromosome [49]. The software package MapQTL 6.0 [50] was used to detect QTLs which were first analysed by interval mapping (IM). The closest marker at each putative QTL identified using interval mapping was selected as a cofactor and the selected markers were used as genetic background controls in the approximate multiple QTL model (MQM). A logarithm of the odds (LOD) threshold value of 3.0 was applied to declare the presence of a QTL at 95% significance level. To determine the effects of other traits on the QTLs for drought and salinity tolerance, QTL for both drought and salinity tolerance were re-analysed by using various agronomic traits (heading dates and awn length reported by Wang [43]) and physiological traits as covariates. Two LOD support intervals around each QTL were established, by taking the two positions, left and right of the peak, that had LOD values of two less than the maximum [50], after performing restricted MQM mapping which does not use markers close to the QTL. The percentage of variance explained by each QTL (R^2^) was obtained using restricted MQM mapping implemented with MapQTL6.0. Graphical representation of linkage groups and QTL was carried out using MapChart 2.2 [51].

### Genomic analysis of potential genes for drought tolerance

The closest marker of the QTL for drought tolerance on 5H is bpb-3241. The sequence of bpb-3241 was downloaded from the website (http://www.diversityarrays.com) and then used to identify the corresponding morex_contig by blast search on the website (http://webblast.ipk-gatersleben.de/barley/). A morex_contig_8158 was found to be homologous with bpb-3241 (Identities = 539/574, 93%). The physical map position of this contig was located at 122.57 cM on 5H. In addition, barley genomic data and gene annotations were downloaded from ftp://ftpmips.helmholtz-muenchen.de/plants/barley/public_data/ [52] and ftp://ftpmips.helmholtz-muenchen.de/plants/barley/public_data/ [53]. Annotated genes around 122.57 cM (118.75-128.54 cM) were examined for potential genes for drought tolerance (Additional file 2: Table S1).

## Results

### Drought, salinity tolerance of the DH lines and proline contents under different stresses

**Figure 1:**
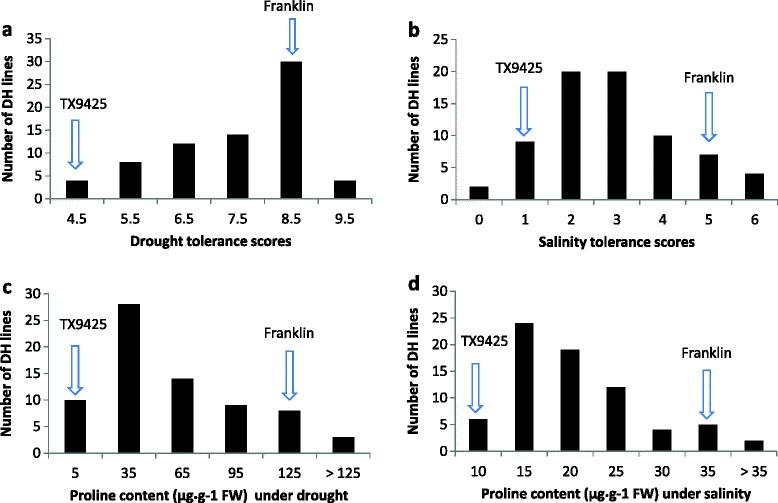
**Frequency distribution for drought (a), salinity (b) tolerance and proline content under drought (c), salinity (d) stress of DH lines derived from the cross of TX9425/Franklin.**

DH lines from the cross between TX9425 and Franklin showed significant difference in drought or salinity tolerance and proline content (P < 0.01) (Additional file 3: Table S2). Figure 1 shows the frequency distribution of drought tolerance (DT) based on leaf wilting, salinity tolerance (ST) based on plant survival scores, and proline content under drought (PC-D) and salinity (PC-S) stress for 72 lines. Continuous distributions were found for all the traits with wilting scores ranging from 4 – 9 for DT, 0–6 for ST, 1.2 -229.9 for PC-D and 7.1 - 49.6 for PC-S. Transgressive segregation was found for all three traits.

### QTL for different traits

**Figure 2:**
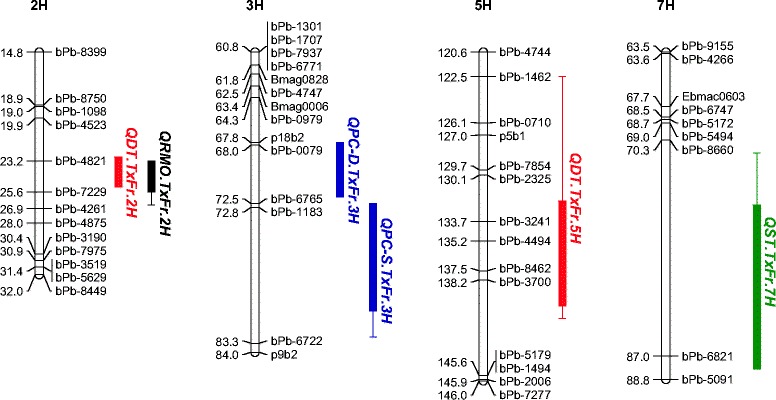
**QTL associated with drought tolerance (in red), salinity tolerance (in green), relative moisture content (black) and proline content under drought or salinity stress (in blue).** For greater clarity, only parts of chromosome regions were shown.

**Figure 3:**
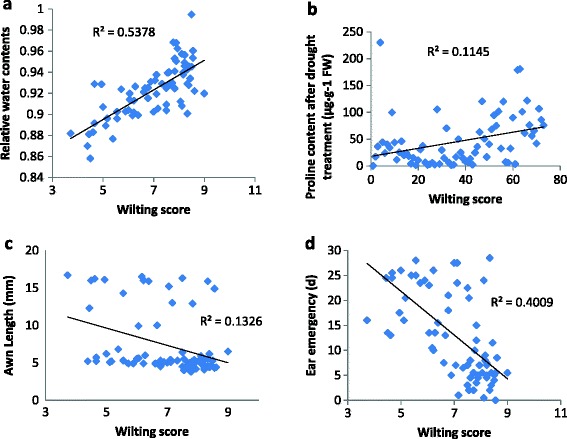
**Correlation analysis. (a)** correlation between RWC (relative water content) and drought tolerance (wilting score), **(b)** correlation between proline content (under drought stress) and drought tolerance, **(c)** correlation between AL (awn length) and drought tolerance, **(d)** correlation between EE (ear emergency) and drought tolerance.

Two QTL for drought tolerance were identified on chromosomes 2H and 5H which were denoted as *QDT.TxFr.2H* and *QDT.TxFr.5H,* respectively (Figure 2, Table 1). bPb-7229 is the nearest marker for *QDT.TxFr.2H*, explaining 42.2% of phenotypic variation. *QDT.TxFr.5H* explained 14.0% of phenotypic variation, with bPb-3700 being the closest marker. Relative water content showed a very close correlation (r = 0.73, P < 0.01) with drought tolerance (wilting scores) (Figure 3a). One QTL (*QRMO.TxFr.2H)* for RWC was identified on a similar position to *QDT.TxFr.2H* on 2H, and it explained 44.3% of phenotypic variation. bPb-7229 is also the closest marker for this QTL. One QTL for proline content under drought conditions was found on 3H, explaining 32.0% of the phenotypic variation. This QTL was at a different position to that for drought tolerance, indicating that drought tolerance and proline production under drought stress was controlled by a different gene(s). This is further confirmed by correlation analysis, that the changes of proline content under drought treatment showed no significant correlation with drought tolerance (Figure 3b).

**Table 1:** **QTLs for agronomic traits detected in the DH population of TX9425 × Franklin (average values)**

**Traits**	**QTL**	**Linkage group**	**Nearest marker**	**Position (cM)**	**LOD**	**R** ^**2**^ **(%)**
DT	QDT.TxFr.2H	2H	bpb-4821	24. 2	8.56	42.2
	QDT.TxFr.5H	5H	bpb-3241	133.7	4.13	14
ST	QST.TxFr.7H	7H	bpb-6821	82.3	5.4	29.2
RWC	QRMO.TxFr.2H	2H	bpb-7229	25. 2	9.45	45.4
PC-D	QPC-D.TxFr.3H	3H	bpb-0079	70.0	6.65	34.7
PC-S	QPC-S.TxFr.3H	3H	bpb-6765	74.8	3.22	18.6

DT: drought tolerance; ST: salinity tolerance; RWC: relative water content; PC-D: proline content under drought tolerance; PC-S: proline content under salinity tolerance.

Only one significant QTL *QST.TxFr.7H* controlling salinity tolerance (estimated by plant survival under salt stress) was found on 7H with a nearest marker bPb-6821. It explained 28.2% of phenotypic variation with a LOD value of 5.17 (Figure 2, Table 1). Under salinity stress, some lines showed a significant increase in proline content. A QTL was identified for proline content on 3H, located on a similar position of the QTL for proline content under drought stress.

### The effect of development genes on QTL for drought tolerance

The performance of agronomic traits under a similar condition to the experiments for both salinity and drought tolerance has been reported in a previous publication [43] and among these different agronomic traits, awn length (AL) showed co-segregation with *uzu* gene [54]. As shown in Table 2 and Figure 3, *PC-D, RWC* and development genes (*uzu* gene and genes for ear emergency) showed significant correlation with drought tolerance. To further confirm the relationships between different traits, a QTL analysis was conducted by using different traits as covariates.

**Table 2:** **Correlation coefficients between different traits**

	**DT**	**RWC**	**PC-D**	**ST**	**PC-S**	**Na** ^**+**^ **content**	**EE**
RWC	0.73						
PC-D	−0.01	−0.13					
ST	−0.47	−0.35	0.01				
PC-S	−0.07	−0.10	0.15	0.37			
Na^+^ content	−0.45	−0.31	0.25	0.10	−0.27		
EE	−0.63	−0.51	−0.31	0.33	−0.10	0.51	
AL	−0.36	−0.43	0.52	0.39	0.21	0.07	−0.02

Significance level: r_0.05_ = 0.23; r_0.01_ = 0.30.

DT: drought tolerance; ST: salinity tolerance; RWC: relative water content; PC-D: proline content under drought tolerance; PC-S: proline content under salinity tolerance; EE: ear emergency; AL: awn length.

**Figure 4:**
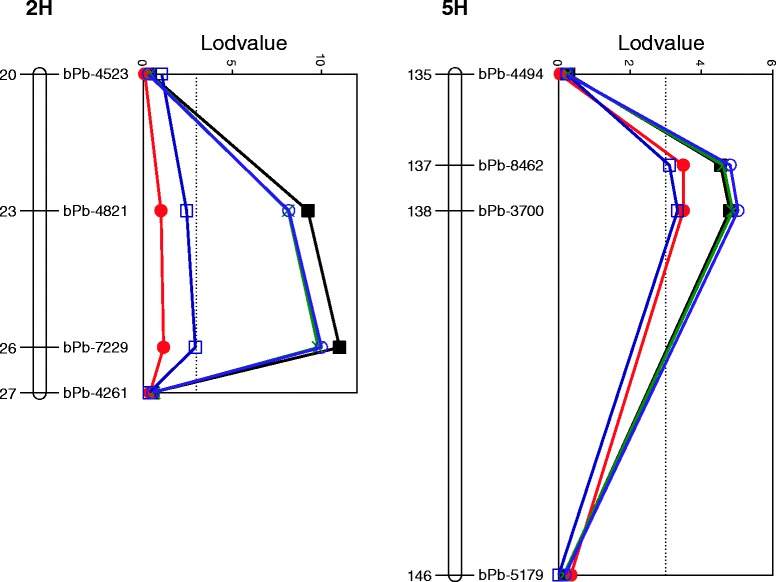
**QTLs associated with drought tolerance (LOD values) on 2H and 5H.** Black solid block: LOD value of original QTL; Purple hollow circle: LOD value of QTL when awn length was used as covariate; Green cross: LOD value of QTL when proline content was used as covariate; Red solid circle: LOD value of QTL when awn length and heading date were used as covariate; Blue hollow block: LOD value of QTL when heading date was used as covariate.

Table 3 lists QTL analysis for drought tolerance by using different traits as covariates. Of the two QTL for drought tolerance, *QDT.TxFr.5H* was less effected, which showed only slight reduction in R^2^ when using RWC and EE as covariates. In contrast, *QDT.TxFr.2H* was significantly affected by genes controlling ear emergency. The QTL, which is located on a similar position to that for RWC and one of the QTL for ear emergency, became insignificant when using either EE or RWC as a covariate. A new QTL for drought tolerance was identified on 3H when using EE as a covariate. This QTL was dependent on the *uzu* gene as it disappeared when AL was also used as a covariate. As expected, proline content under drought treatment showed little effects on R^2^ of both QTL for drought tolerance (Figure 4).

**Table 3:** **QTL for drought tolerance when different physiological and developmental traits were used as covariates**

**QTL**	**Covariate**	**Linkage group**	**Nearest marker**	**Position (cM)**	**LOD**	**R** ^**2**^ **(%)**
QDT.TxFr.2H	Heading date				ns	ns
QDT.TxFr.3H		3H	bpb-0079	67.3	4.5	10.9
QDT.TxFr.5H		5H	bpb-3241	133.7	3.51	8
QDT.TxFr.2H	Awn length	2H	bpb-4821	24. 2	10.0	35.6
QDT.TxFr.5H		5H	bpb-3241	133.7	5.03	15.1
QDT.TxFr.2H	Awn Length + heading date				ns	ns
QDT.TxFr.5H		5H	bpb-3241	133.7	3.51	8.2
QDT.TxFr.2H	Proline	2H	bpb-4821	24. 2	9.82	38.0
QDT.TxFr.5H		5H	bpb-3241	133.7	4.88	15.9

ns: not significant.

### The effect of PC-S, RWC, Na^+^ content and development genes on QTL for salinity tolerance

*PC-S, RWC* and development genes (*uzu* gene and genes for ear emergency) also showed significant correlation with salinity tolerance (Table 2). However, when a QTL analysis was conducted by using these traits as covariates, very minor effects were shown with the percentage of the phenotypic variation determined by the QTL (R^2^) being reduced from 28% to around 20%, indicating that the tolerance gene is most likely independent of these traits. Na^+^ content showed relatively low correlation (Table 2) with salinity tolerance, which is consistent with the above results.

### The effect of development genes on QTL for physiological traits

Developmental genes showed huge effects on the QTL for proline. No significant QTL were detected for proline contents under both drought and salinity stress when using awn length as a covariate.

Even though QTL for drought tolerance and salinity tolerance were located on different chromosomes, the R^2^ of the QTL for salinity tolerance was reduced from 28% to around 16% when using drought tolerance scores as a covariate. Likewise, the R^2^ of the QTL for drought tolerance on 2H was reduced from 44% to around 32% when using salinity tolerance scores as a covariate.

### Potential genes for drought tolerance on 5H

Two QTL were identified for drought tolerance. Since the QTL on 2H was closely linked to development gene, the searching for candidate genes for drought tolerance was only conducted for the QTL on 5H. Among all annotated genes, a gene (MLOC_18300.1) coding 9-cis-epoxycarotenoid dioxygenase 2 (Additional file 2: Table S1), which is close to the marker bpb-3241 according to the PopSeq map [53], is most likely the candidate gene for this QTL. 9-cis-epoxycarotenoid dioxygenase 2 is an important enzyme during ABA synthesis under drought stress. Some other genes for transcriptional factors such as WRKY, ARF were found in this region and may also be involved in abiotic stress.

## Discussion

### QTL on 5H for drought tolerance is less affected by plant height and maturity

Drought is one of the major abiotic yield-limiting factors in crops which have been affected by early season water deficit worldwide. Therefore, understanding the genetic background and enhancing drought tolerance is crucial for both breeding and basic research. Owing to the complexity of drought, strong QTL-environment interaction, possible epistatic effects and small explanation of drought tolerance loci, the knowledge on drought tolerance is still incomplete [39,55]. In barley, QTL analysis for numerous traits has been performed under drought stress or Mediterranean rainfed conditions including: leaf wilting [36], proline content [36], chlorophyll content [56], relative water content [10,39,57], osmotic adjustment [39], carbon isotope discrimination [58], water-soluble carbohydrate concentration (WSC) [39,57], flowering time or heading date [59,60], plant height [42,60], grain yield and seed quality parameters [37,59]. However, most of them are not dealing with the drought tolerance which is the changes of the traits under drought conditions compared to controls. In this study, we used leaf wilting as a major index for drought tolerance (no wilting was shown in controls) and identified two QTLs controlling drought tolerance on 2H (*QDT.TxFr.2H*) and 5H (*QDT.TxFr.5H*). These QTL were different from those reported by Sayed et al. [36] who also used leaf wilting as one of the criteria for drought tolerance. The co-localization of *QDT.TxFr.2H* and another QTL for relative moisture (*QRMO.TxFr.2H*) suggested a common genetic control between them and the possibility for RMO under drought stress used as selection criteria for drought tolerance. However, *QDT.TxFr.2H* and *QRMO.TxFr.2H* were located in the similar position as a QTL conferring heading date on 2H which was identified by Wang et al. [43]. When QTLs for heading date and awn length [43] was added to the analysis as covariates, *QDT.TxFr.2H* could not be detected (Table 3), indicating that these two traits were dependent on the development genes. Even though no association was found between drought tolerance and heading date in one of the reports [61], drought escape via a short life cycle, together with drought avoidance, drought tolerance and drought recovery are crucial mechanisms of drought resistance. Under drought stress, early flowering is a beneficial trait for plants to escape from stress at the expense of reduced yield potential [62]. In contrast to *QDT.TxFr.2H, QDT.TxFr.5H* was less affected by different development genes (Table 2). Thus *QDT.TxFr.5H* could be a candidate locus for further drought tolerance study. 9-cis-epoxycarotenoid dioxygenase 2 which has key roles in the synthesis of ABA under drought stress [63] could be one of the candidate genes for this QTL. Overexpression of 9-cis-epoxycarotenoid dioxygenase gene increases ABA levels and enhances drought tolerance [64]. The development of near isogenic lines based on this locus should be the best approach to avoid the interference of other development genes and to fine map this QTL.

### Salinity tolerance identified from this population was not linked with Na^+^ absorption

Salinity tolerance is controlled by multi-gene traits where genes are expressed at various plant developmental stages. A large number of agronomic and physiological indices were used to quantify plant salinity stress tolerance including: seed germination [65], plant survival [15,40], Na^+^ exclusion [14], tissue ion content [66], yield and agronomic traits [66,67], chlorophyll content and water soluble carbohydrate [68]. In the current experiment, plant survival under saline conditions was scored at seedling stage and one major QTL for salinity tolerance (*QST.TxFr.7H*) was identified on chromosome 7H (Figure 2). This QTL was at a similar position to the one (*QST.YyFr.7H*) recently identified by Zhou et al. [40] and another trait HvNax3 on 7H controlling Na^+^ exclusion identified by Shavrukov et al. [14]. However, in this population, leaf Na^+^ content showed no correlation with salinity tolerance. The most likely explanation for this is that in the above studies plants were treated with much lower levels of NaCl. Under these conditions, plants were able to osmotically adjust to relatively mild hyperosmotic stress by *de novo* synthesis of compatible solutes and, hence, did not rely on the use of Na^+^. In our work, 320 mM NaCl was used to screen plants. Osmotic adjusting to this stress by *de novo* synthesis of compatible solutes would come at a huge metabolic cost [69], and Na^+^ uptake into the leaf was an energetically more favourable option (on a provision it is effectively sequestered in the vacuole). As leaf Na^+^ analysis for QTL mapping was done at the whole-tissue level and did not differentiate between the Na^+^ distribution between the cytosol and the vacuole, the lack of correlation between Na^+^ content and salt tolerance is hardly surprising.

### The changes of proline content under drought and salinity stresses are not necessarily linked to drought and salinity tolerance evaluated using the methods in this experiment

Under control conditions, proline is needed to participate in normal metabolisms and regulate plant developmental processes [70]. Various abiotic stresses can induce proline biosynthesis [17] to balance osmotic pressure in cells, maintain redox balance and activate signalling networks for stress adaption [70]. In the current study, proline levels increased in plants exposed to both drought and salinity stress. QTLs for proline contents under drought (*QPC-D.TxFr.3H*) and salinity stress (*QPC-S.TxFr.3H*) were identified to be at similar positions. A QTL was also found on 3H in a previous report but at a different position according to consensus maps [71]. However, they were at different positions with QTLs for either drought (*QDT.TxFr.2H*, *QDT.TxFr.5H*) or salinity stress (*QST.TxFr.7H*) tolerance (Figure 2, Table 1). QTL analysis for drought and salinity tolerance using proline content as a covariate further confirmed that there was no correlation between proline accumulation and tolerance to either stress. The results suggested that proline biosynthesis under drought or salinity stresses is not necessarily linked to drought or salinity tolerance. As commented above, the high metabolic cost of proline biosynthesis may be the reason.

Interestingly, QTL conferring proline content under abiotic stress were at the similar position to the QTL for awn length on chromosome 3H with bpb-0079 as closest marker [43,54]. As shown in Table 2, QPC-D.TxFr.3H had disappeared after adding awn length as covariate for QTL analysis, indicating that proline biosynthesis may have some cross-talks with plant development. Increasing data from over-expressions or knock-out mutants of proline synthesis genes indicate that proline participates in embryo and plant development [72], influences leaf or inflorescences morphology [73] and affects blossoms time [74]. In this experiment, proline biosynthesis was most likely involved in plant height instead of maturity. This was confirmed by further QTL analysis using plant height and heading date as covariates. The percentage of phenotypic variation determined by the QTL for proline was significantly reduced when using plant height as covariate but not affected when using heading date as covariate (data not shown).

### The effect of population size, marker distortion and phenotyping accuracy on the estimation of QTL

A limited population size used in QTL detection may lead to underestimation of QTL number, overestimation of QTL effects. The number of detected QTL increased with increased population size but the most of increased QTL were with small effects [75]. In our experiment, most of the QTL for various traits determined a larger percent of phenotypic variation even though some of the LOD values were just above the significance level of 3.0. Even though a large number of markers were used for map construction of this population, the markers were not evenly distributed and many markers showed significant distortion. Missing markers and marker distortion can also affect the accuracy of QTL detection [76]. In this experiment, the QTL for salinity tolerance on 2H detected in a different population with the same salinity tolerant variety [15] was not identified in this population. An extra experiment was conducted and further confirmed that there was no QTL on 2H for salinity tolerance (data not shown). Further studies are needed to see if it’s due to population size or missing markers. For the QTL on 5H for drought tolerance, we are constructing near-isogenic lines which are needed for the fine mapping of this QTL.

Accurate phenotyping is also crucial in locating QTL controlling quantitative traits [77]. Most of the traits reported in this paper showed relatively small variation between different experiments. However, proline contents under drought condition showed a huge variation between replications (Additional file 3: Table S2). This will limit the use of this trait in breeding programs.

## Conclusion

QTL mapping approach was used in this study to determine the linkages between stress tolerance and different physiological and developmental traits. A QTL on 5H for drought tolerance was less affected by other developmental traits and this locus can be effectively used in breeding programs.

## Additional files

Additional file 1: Figure S1. Drought tolerance of different DH lines. **A**: Experiment I and II (left: tolerant – a score of 1; middle: sensitive – a score of 9; right: medium tolerant – a score of 4); **B**: Experiment III (left sensitive – a score of 8; right tolerant – a score of 1). Additional file 2: Table S1. List of genes close to bPb-3241 on chromosome 5H. Additional file 3: Table S2. Average performance of different traits with standard deviation.

## Abbreviations

QTL: Quantitative trait loci
RWC: Relative water content
P5CS: Pyrroline-5-carboxylate synthetase
P5CR: Pyrroline-5-carboxylate reductase
ROS: Reactive oxygen species
DH: Doubled-haploid
SA: Sulphosalicylic acid
IM: Interval mapping
MQM: Multiple QTL model
LOD: Logarithm of the odds
DT: Drought tolerance
ST: Salinity tolerance
PC-D: Proline content under drought stress
PC-S: Proline content under salinity stress
RMO: Relative moisture
AL: Awn length
EE: Ear emergency
WSC: Water-soluble carbohydrate concentration
