# Supplementary material for: Using QTL mapping to investigate the relationships between abiotic stress tolerance (drought and salinity) and agronomic and physiological traits
Source: BMC Genomics. 2015 Feb 5;16(1):43. doi: 10.1186/s12864-015-1243-8 (PMC4320823; doi:10.1186/s12864-015-1243-8)
Supplement: Additional file 1: Figure S1. — Drought tolerance of different DH lines. A: Experiment I and II (left: tolerant – a score of 1; middle: sensitive – a score of 9; right: medium tolerant – a score of 4); B: Experiment III (left sensitive – a score of 8; right tolerant – a score of 1). [file 12864_2015_1243_MOESM1_ESM.pdf]

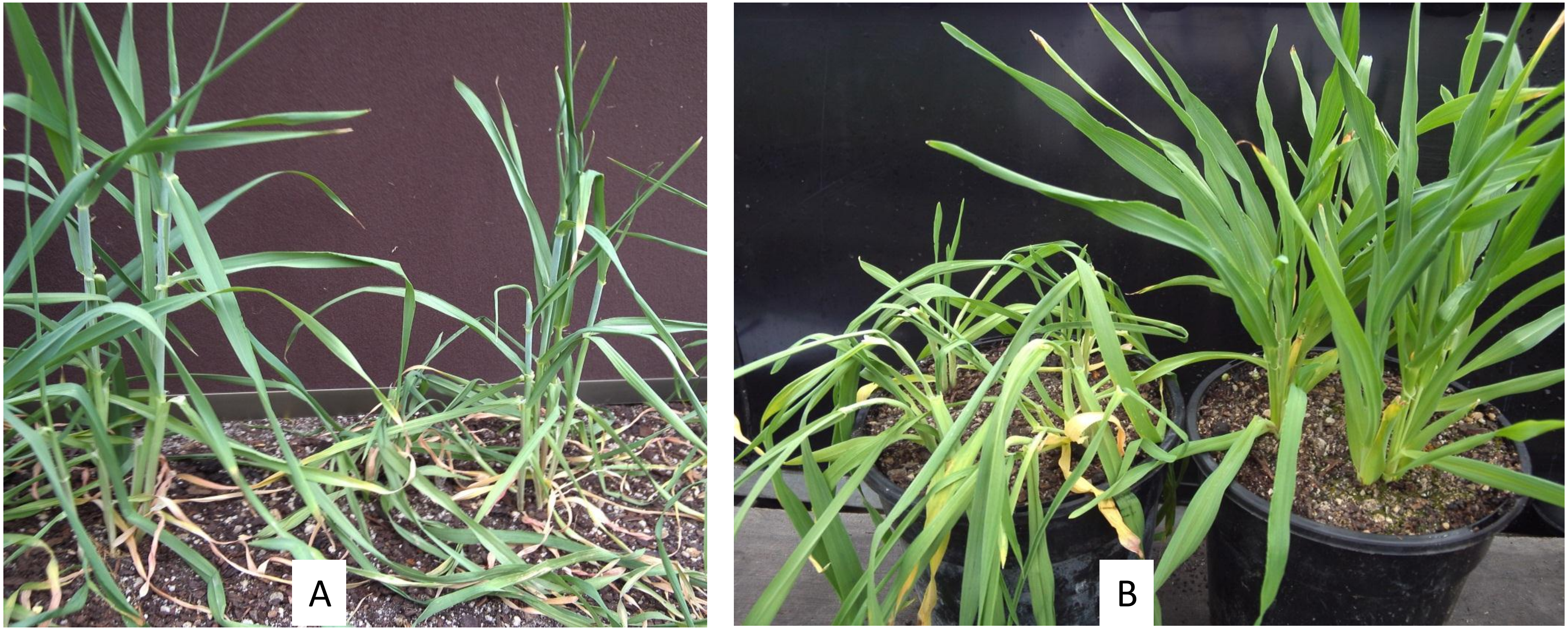

Fig. S1. Drought tolerance of different DH lines. A: Experiment I and II (left: tolerant – a score of 1; middle: sensitive – a score of 9; right: medium tolerant – a score of 4); B: Experiment III (left sensitive – a score of 8; right tolerant – a score of 1).
