# Supplementary material for: Using QTL mapping to investigate the relationships between abiotic stress tolerance (drought and salinity) and agronomic and physiological traits
Source: BMC Genomics. 2015 Feb 5;16(1):43. doi: 10.1186/s12864-015-1243-8 (PMC4320823; doi:10.1186/s12864-015-1243-8)
Supplement: Additional file 3: Table S2. — Average performance of different traits with standard deviation. [file 12864_2015_1243_MOESM3_ESM.docx]

Table S2 Average performance of different traits with standard deviation

| DH Line | Drought tolerance | Relative water content | Proline content under drought | Salinity tolerance | Proline content under salinity | Na^+^ content under salinity |
| --- | --- | --- | --- | --- | --- | --- |
| TF003 | 8.11 ± 1.54 | 0.957 ± 0.037 | 16.95 ± 2.8 | 0.00 ± 0.00 | 18.7 ± 3.0 | 67.2 ± 8.7 |
| TF004 | 5.17 ± 2.17 | 0.896 ± 0.054 | 36.2 ± 49.6 | 0.50 ± 0.50 | 13.2 ± 0.1 | 89.9 ± 0.2 |
| TF008 | 7.17 ± 1.48 | 0.905 ± 0.019 | 229.9 ± 92.3 | 1.17 ± 0.96 | 23.9 ± 10.4 | 79.7 ± 57.7 |
| TF010 | 6.61 ± 1.78 | 0.921 ± 0.008 | 44.3 ± 14.6 | 2.17 ± 0.96 | 15.3 ± 2.4 | 75.4 ± 11.0 |
| TF012 | 8.17 ± 2.02 | 0.940 ± 0.033 | 23.85 ± 26.4 | 3.83 ± 1.57 | 29.8 ± 29.7 | 83.1 ± 4.2 |
| TF014 | 5.56 ± 2.12 | 0.896 ± 0.080 | 40.4 ± 56.4 | 4.17 ± 0.69 | 7.6 ± 5.5 | 66.8 ± 4.5 |
| TF015 | 7.00 ± 1.45 | 0.902 ± 0.074 | 33.9 ± 47.5 | 2.17 ± 1.50 | 9.3 ± 2.7 | 88.0 ± 7.1 |
| TF016 | 5.39 ± 0.35 | 0.877 ± 0.061 | 99.7 ± 121.5 | 4.17 ± 1.34 | 11.5 ± 1.6 | 68.7 ± 7.2 |
| TF019 | 3.72 ± 0.86 | 0.882 ± 0.079 | 43.55 ± 40.7 | 3.33 ± 1.94 | 20.5 ± 2.9 | 88.3 ± 6.0 |
| TF020 | 8.44 ± 1.90 | 0.934 ± 0.053 | 11.6 ± 4.2 | 4.00 ± 1.15 | 34.9 ± 17.4 | 89.9 ± 36.8 |
| TF022 | 4.67 ± 1.76 | 0.892 ± 0.080 | 25.35 ± 7.4 | 4.50 ± 1.15 | 20.0 ± 12.2 | 76.7 ± 6.2 |
| TF023 | 6.39 ± 2.68 | 0.899 ± 0.059 | 46.05 ± 42.5 | 5.50 ± 0.50 | 31.6 ± 2.5 | 76.9 ± 7.3 |
| TF025 | 5.89 ± 2.52 | 0.903 ± 0.087 | 19.8 ± 25.5 | 0.17 ± 0.50 | 17.3 ± 6.8 | 64.0 ± 7.3 |
| TF026 | 6.89 ± 2.52 | 0.931 ± 0.031 | 23.85 ± 31.5 | 1.33 ± 0.88 | 20.3 ± 5.1 | 74.0 ± 5.0 |
| TF028 | 8.06 ± 1.23 | 0.912 ± 0.066 | 18 ± 24.0 | 2.00 ± 0.58 | 17.8 ± 5.0 | 49.1 ± 16.6 |
| TF030 | 8.39 ± 2.07 | 0.928 ± 0.045 | 4.2 ± 5.2 | 2.83 ± 0.83 | 14.6 ± 3.9 | 45.8 ± 12.7 |
| TF031 | 6.28 ± 2.56 | 0.926 ± 0.040 | 15 ± 14.7 | 4.00 ± 1.00 | 12.1 ± 3.8 | 72.4 ± 5.0 |
| TF033 | 7.11 ± 3.27 | 0.919 ± 0.076 | 1.25 ± 0.5 | 4.33 ± 1.37 | 32.9 ± 22.7 | 66.4 ± 5.1 |
| TF034 | 7.94 ± 1.11 | 0.962 ± 0.000 | 10.65 ± 12.9 | 2.00 ± 1.00 | 12.1 ± 6.8 | 81.6 ± 7.4 |
| TF035 | 5.11 ± 2.46 | 0.889 ± 0.067 | 30.45 ± 21.4 | 1.83 ± 1.17 | 14.0 ± 3.2 | 79.5 ± 3.9 |
| TF039 | 4.39 ± 2.00 | 0.870 ± 0.066 | 37.5 ± 9.2 | 4.50 ± 1.26 | 21.6 ± 12.1 | 90.5 ± 2.9 |
| TF040 | 7.61 ± 2.58 | 0.929 ± 0.053 | 6.65 ± 1.8 | 3.67 ± 1.25 | 9.9 ± 4.5 | 65.0 ± 9.8 |
| TF042 | 7.56 ± 3.40 | 0.939 ± 0.034 | 1.7 ± 1.3 | 1.83 ± 0.69 | 15.1 ± 2.5 | 76.4 ± 4.5 |
| TF049 | 8.22 ± 2.52 | 0.949 ± 0.014 | 3.4 ± 1.3 | 1.33 ± 1.15 | 11.5 ± 5.7 | 63.0 ± 3.4 |
| TF050 | 8.56 ± 1.71 | 0.960 ± 0.021 | 5.75 ± 1.2 | 0.33 ± 0.33 | 12.1 ± 10.4 | 45.0 ± 4.2 |
| TF055 | 6.22 ± 2.80 | 0.913 ± 0.083 | 2.4 ± 2.8 | 2.17 ± 1.42 | 10.2 ± 2.6 | 83.9 ± 1.0 |
| TF056 | 7.83 ± 1.92 | 0.941 ± 0.030 | 105.25 ± 82.1 | 5.17 ± 1.29 | 49.6 ± 49.3 | 42.5 ± 29.6 |
| TF057 | 5.72 ± 2.15 | 0.924 ± 0.073 | 3.6 ± 4.1 | 3.67 ± 1.41 | 7.3 ± 2.2 | 60.2 ± 14.4 |
| TF058 | 9.00 ± 1.20 | 0.920 ± 0.039 | 29.6 ± 7.8 | 0.83 ± 0.69 | 21.2 ± 5.0 | 55.3 ± 15.8 |
| TF062 | 6.06 ± 2.95 | 0.914 ± 0.064 | 14.55 ± 19.9 | 2.83 ± 1.45 | 45.1 ± 68.3 | 66.5 ± 4.3 |
| TF065 | 8.33 ± 1.15 | 0.901 ± 0.050 | 70.3 ± 49.3 | 2.00 ± 0.82 | 14.3 ± 10.1 | 40.1 ± 19.3 |
| TF070 | 5.00 ± 2.03 | 0.907 ± 0.077 | 6.4 ± 4.2 | 4.00 ± 1.73 | 8.5 ± 4.7 | 65.7 ± 6.1 |
| TF071 | 8.22 ± 3.08 | 0.956 ± 0.000 | 1.2 ± 0.1 | 2.33 ± 1.37 | 19.1 ± 3.1 | 71.6 ± 15.2 |
| TF074 | 8.33 ± 2.89 | 0.943 ± 0.042 | 2.1 ± 2.4 | 0.83 ± 0.96 | 11.7 ± 4.9 | 68.8 ± 10.1 |
| TF081 | 8.33 ± 2.08 | 0.929 ± 0.031 | 16.55 ± 20.6 | 2.67 ± 0.58 | 11.7 ± 10.3 | 53.5 ± 9.1 |
| TF082 | 7.50 ± 0.83 | 0.909 ± 0.041 | 49.9 ± 63.1 | 2.33 ± 0.88 | 13.6 ± 8.5 | 78.7 ± 10.0 |
| TF083 | 8.17 ± 1.89 | 0.938 ± 0.040 | 24.9 ± 6.5 | 2.17 ± 0.96 | 20.8 ± 4.8 | 78.7 ± 1.7 |
| TF084 | 7.50 ± 2.18 | 0.925 ± 0.030 | 13.9 ± 10.5 | 1.00 ± 1.00 | 14.9 ± 3.4 | 54.2 ± 13.2 |
| TF085 | 5.56 ± 1.95 | 0.902 ± 0.080 | 8 ± 8.9 | 3.50 ± 1.50 | 17.3 ± 5.7 | 78.3 ± 7.8 |
| TF088 | 7.89 ± 1.92 | 0.950 ± 0.024 | 7.6 ± 9.6 | 2.50 ± 1.50 | 15.4 ± 1.6 | 76.6 ± 11.9 |
| TF091 | 6.78 ± 2.71 | 0.925 ± 0.043 | 13.55 ± 15.5 | 1.83 ± 1.45 | 13.7 ± 2.6 | 85.6 ± 14.4 |
| TF098 | 7.11 ± 2.99 | 0.938 ± 0.058 | 1.9 ± 2.0 | 2.83 ± 1.17 | 7.1 ± 5.0 | 76.9 ± 22.6 |
| TF100 | 8.61 ± 0.35 | 0.922 ± 0.026 | 24.55 ± 26.5 | 2.33 ± 0.88 | 17.1 ± 3.5 | 68.3 ± 6.2 |
| TF101 | 7.89 ± 1.64 | 0.968 ± 0.024 | 35.8 ± 35.9 | 1.17 ± 0.69 | 20.8 ± 5.2 | 57.1 ± 16.5 |
| TF102 | 4.44 ± 2.22 | 0.881 ± 0.067 | 63.1 ± 88.0 | 3.67 ± 1.67 | 33.8 ± 16.0 | 85.1 ± 7.8 |
| TF105 | 4.50 ± 1.83 | 0.858 ± 0.057 | 120.2 ± 137.2 | 3.33 ± 1.60 | 28.2 ± 11.8 | 76.3 ± 7.6 |
| TF106 | 4.61 ± 2.37 | 0.883 ± 0.078 | 50.6 ± 20.2 | 5.67 ± 0.58 | 26.6 ± 6.7 | 91.4 ± 3.6 |
| TF108 | 7.83 ± 1.61 | 0.953 ± 0.031 | 16.55 ± 17.0 | 4.83 ± 1.89 | 31.2 ± 24.9 | 99.5 ± 14.0 |
| TF109 | 8.39 ± 1.51 | 0.946 ± 0.020 | 40.45 ± 11.1 | 1.33 ± 1.15 | 20.5 ± 3.7 | 88.8 ± 38.7 |
| TF112 | 6.22 ± 2.34 | 0.902 ± 0.033 | 68.05 ± 91.7 | 1.50 ± 0.96 | 18.9 ± 4.2 | 79.9 ± 13.1 |
| TF116 | 6.17 ± 1.92 | 0.899 ± 0.033 | 94.6 ± 132.7 | 2.17 ± 1.57 | 11.0 ± 2.0 | 92.6 ± 5.8 |
| TF117 | 7.33 ± 1.86 | 0.929 ± 0.010 | 75 ± 104.1 | 2.00 ± 0.82 | 17.4 ± 1.8 | 80.9 ± 6.5 |
| TF123 | 7.50 ± 0.50 | 0.904 ± 0.009 | 101.4 ± 92.6 | 1.67 ± 1.11 | 23.5 ± 3.1 | 41.7 ± 6.2 |
| TF125 | 8.22 ± 1.95 | 0.946 ± 0.015 | 32.35 ± 12.8 | 0.50 ± 0.50 | 22.4 ± 1.3 | 49.0 ± 4.2 |
| TF126 | 8.50 ± 1.88 | 0.945 ± 0.033 | 119.95 ± 113.6 | 1.50 ± 1.26 | 16.1 ± 7.9 | 53.1 ± 5.9 |
| TF127 | 7.78 ± 3.02 | 0.968 ± 0.037 | 7.15 ± 8.4 | 0.67 ± 0.58 | 13.0 ± 3.3 | 55.0 ± 2.9 |
| TF128 | 7.83 ± 3.03 | 0.925 ± 0.042 | 5.7 ± 6.4 | 0.67 ± 0.67 | 15.8 ± 1.8 | 69.0 ± 9.8 |
| TF129 | 6.00 ± 1.45 | 0.904 ± 0.054 | 32.4 ± 42.4 | 2.83 ± 1.26 | 11.1 ± 3.0 | 65.2 ± 9.7 |
| TF133 | 8.11 ± 1.26 | 0.908 ± 0.035 | 90 ± 125.2 | 1.67 ± 0.83 | 13.3 ± 7.6 | 53.8 ± 3.2 |
| TF134 | 4.67 ± 0.67 | 0.929 ± 0.061 | 3.6 ± 4.0 | 2.83 ± 1.07 | 12.6 ± 4.1 | 73.2 ± 12.0 |
| TF135 | 8.56 ± 1.95 | 0.954 ± 0.032 | 178.7 ± 132.5 | 3.00 ± 0.82 | 18.5 ± 5.7 | 32.1 ± 6.1 |
| TF138 | 7.06 ± 2.65 | 0.932 ± 0.031 | 180.55 ± 222.7 | 2.83 ± 0.69 | 15.2 ± 6.3 | 42.4 ± 12.0 |
| TF139 | 6.67 ± 3.53 | 0.925 ± 0.006 | 100.95 ± 137.7 | 1.17 ± 0.96 | 13.2 ± 3.0 | 70.8 ± 3.4 |
| TF140 | 6.06 ± 1.44 | 0.920 ± 0.047 | 61.1 ± 77.9 | 5.00 ± 0.58 | 28.6 ± 18.9 | 55.1 ± 7.8 |
| TF145 | 8.22 ± 0.69 | 0.957 ± 0.040 | 121.85 ± 168.9 | 1.50 ± 0.50 | 13.1 ± 4.5 | 37.6 ± 7.5 |
| TF146 | 8.50 ± 0.50 | 0.995 ± 0.024 | 76 ± 23.3 | 1.17 ± 0.17 | 13.8 ± 8.0 | 57.1 ± 11.5 |
| TF148 | 6.78 ± 0.69 | 0.915 ± 0.017 | 55.9 ± 73.3 | 2.50 ± 0.96 | 17.7 ± 6.7 | 61.4 ± 7.1 |
| TF149 | 7.72 ± 1.18 | 0.930 ± 0.005 | 65.2 ± 89.8 | 2.67 ± 1.86 | 10.2 ± 2.4 | 39.8 ± 12.7 |
| TF150 | 7.72 ± 2.75 | 0.929 ± 0.009 | 41.85 ± 57.6 | 0.00 ± 0.00 | 15.0 ± 3.8 | 58.3 ± 0.4 |
| TF154 | 7.56 ± 1.07 | 0.926 ± 0.008 | 106.6 ± 145.4 | 1.17 ± 0.96 | 20.2 ± 2.6 | 44.3 ± 6.7 |
| TF158 | 4.94 ± 0.92 | 0.928 ± 0.049 | 86.15 ± 119.6 | 6.00 ± 1.41 | 17.3 ± 9.5 | 77.5 ± 5.4 |
| TF509 | 7.61 ± 2.30 | 0.940 ± 0.023 | 75.75 ± 106.1 | 2.33 ± 1.41 | 21.7 ± 19.1 | 81.8 ± 8.3 |
